# Pregnancy Screening before Diagnostic Radiography in Emergency Department; an Educational Review

**Published:** 2017-02-28

**Authors:** Abdelrahman Ibrahim Abushouk, Morteza Sanei Taheri, Parichehr Pooransari, Sahar Mirbaha, Alaleh Rouhipour, Alireza Baratloo

**Affiliations:** 1 Faculty of Medicine, Ain Shams University, Cairo, Egypt.; 2 Department of Radiology, Shohadaye Tajrish Hospital, Shahid Beheshti University of Medical Sciences, Tehran, Iran.; 3 Department of Ob&Gyn, Shohadaye Tajrish Hospital, Shahid Beheshti University of Medical Sciences, Tehran, Iran.; 4 Department of Emergency Medicine, Shahid Beheshti University of Medical Sciences, Tehran, Iran.; 5 Pediatric Specialist, Private Researcher, Tehran, Iran.; 6 Department of Emergency Medicine, Tehran University of Medical Sciences, Tehran, Iran.

**Keywords:** Radiation, Imaging, Pregnancy, Screening, Emergency Department

## Abstract

In modern medical practice, there is an increasing dependence on imaging techniques in most medical specialties. Radiation exposure during pregnancy may have serious teratogenic effects to the fetus. Therefore, checking the pregnancy status before imaging women of child bearing age can protect against these effects. Lack of international regulations and standard protocols exposes the patient to unexpected fetal radiation effects and the health professionals to medicolegal suits. Recently, the American Academy of Radiology and the European community of Medical Ionizing Radiation Protection released national guidelines regarding pregnancy screening before imaging potentially pregnant females. However, different methods of pregnancy screening exist among different radiology centers. This review aims to discuss the most recent guidelines for imaging females of childbearing age and highlight the need for an international regulation to guide pregnancy screening before diagnostic radiation exposure.

## Introduction

In modern medical practice, there is an increasing dependence on imaging techniques in most medical specialties, especially emergency medicine (1). Radiation exposure during pregnancy may have serious teratogenic effects to the fetus. In 2000, the International Commission on Radiation Protection published a statement, which indicated that thousands of pregnant women are unintentionally exposed to ionizing radiation annually (2). It was reported that 1% of reproductive age females who had an abdominal imaging procedure were pregnant in their first trimester (3-5).

Therefore, checking the pregnancy status before imaging women of child bearing age can protect against radiation teratogenic effects. Lack of international regulations and standard protocols exposes the patients to unexpected radiation effects and the health professionals to medicolegal suits. However, there are different approaches in this regard. Some centers offer a costless urine pregnancy test. Others obtain an informed written consent or a signed questionnaire indicating women's awareness of any imaging risks and acknowledging the lack of a pregnancy probability before radiation exposure (6). Due to lack of resources, some centers acts according to the patient's trust (7).

Despite the absence of a standard regulation to guide radiological testing in these situations, several organizations such as the National Academy of Sciences, National Institute of Environmental Health, and the International Committee on Radiological Protection recommended radiologists to ask women about their pregnancy status and menstrual date before radiological testing (2). 

**Table 1:** Risk of teratogenic malformations according to dose of radiation and gestational age on exposure

**Gestational age (week)**	**Radiation dose**		
	**< 5 rad**	**5 - 10 rad**	**> 10 rad**
**Prior to conception**	None	None	None
**1** ^st^ **-2** ^nd^	None	None	Possible spontaneous abortion
**3** ^rd^ ** - 8** ^th^	None	Subclinical effects	Possible malformation
**9** ^th^ ** - 15** ^th^	None	Subclinical effects	Increased risk of IQ deficits
**16** ^th^ ** - 25** ^th^	None	None	IQ deficits not detectable at diagnostic doses
**>25** ^th^	None	None	None applicable to diagnostic medicine

**Table 2:** Fetal radiation dose correlation with risk of childhood cancer as stated by the Health Protection Agency Centre for Radiation Chemical and Environmental Hazards

**Estimated fetal dose (rad)**	**Radiological examination**	**Risk of childhood cancer**
0.0001-0.001	X-ray (Head and Thoracic spine) CT scan (Head and Neck). Breast Mammography	< 1 in 1,000,000
0.001-0.01	CT pulmonary angiogram Lung ventilation scans	1 in 1,000,000 to 1 in 100,000
0.01-0.1	X-ray (Abdomen, pelvis, hip, and Barium meal) CT scan (Chest and liver)	1 in 100,000 to 1 in 10,000
0.1-1	X-ray (Lumbar spine and Barium enema) CT scan (Abdomen and lumbar spine) Myocardial scan, Bone scan, and Tumor scan	1 in 10,000 to in 1,000
1-5	CT scan (Pelvis and abdomen) Whole body scan	1 in 1,000 to 1 in 200

**Figure 1:**
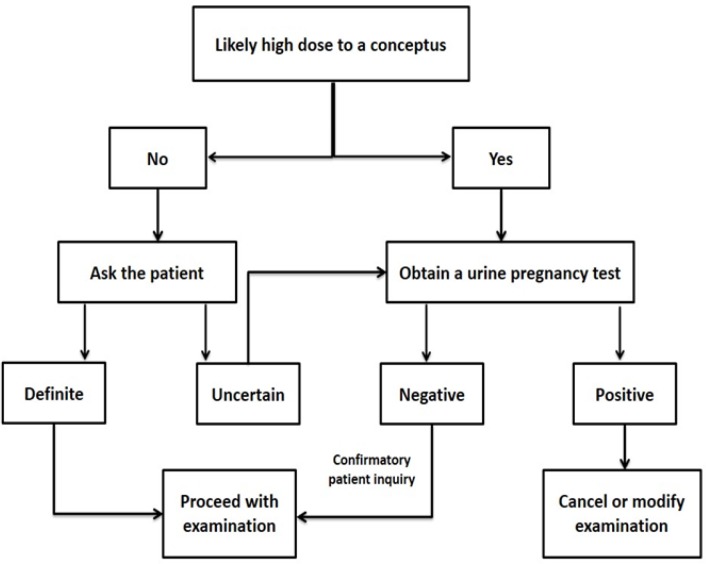
American College of Radiology (ACR) practice guidelines for imaging pregnant and potentially pregnant women

In 2007, Applegate initially clarified the need for such guideline and stated what aspects it should include. According to his statement, this guideline should address the method of screening, documentation of the results, protection of patient's privacy, and situation management in case of emergency radiological testing (7). 

The evidence regarding the physicians' awareness of possible radiation risks to the fetus is controversial. In surveys by Ratnaplan et al., and Bentur et al., physicians had a strong perception of high radiation associated teratogenic risk; therefore, they limited requesting ionizing radiation procedures for pregnant women (8). In another survey by Ikpeme et al., 98.8% of physicians supplied radiological practitioners with lacking information regarding the pregnancy status of reproductive age women, which could be attributed to lack of awareness or underestimation of radiation-induced fetal risks (9).

This review discusses the current literature trend towards pregnancy screening before radiological imaging of reproductive age women and highlights the need to establish an international regulation to guide these diagnostic procedures.

**A) Patients and physicians awareness:**

***- Risks of radiation to the fetus***

As illustrated in table 1, exposure within the first two weeks of conception is only associated with a risk of pregnancy loss at high radiation doses above 0.1 Gy (10 rad). Later exposure until the 15^th^ week is associated with developmental anomalies, only at doses exceeding 0.1 Gy (10). Exposure after the first 15 weeks of pregnancy only poses risk of central nervous system deficits at extremely high doses of more than 0.2 Gy (20 rad) (2, 11). The overall lifetime radiation-induced cancer for an exposed fetus to 0.05 Gy (5 rad) in the period following the 15^th^ week of pregnancy is estimated to be 2% (12, 13). A detailed correlation between the fetal radiation dose and the risk of childhood cancer is illustrated in table 2.

***-Medicolegal issues***

Defining the pregnancy status and supplying radiological practitioners with all relevant information before radiological imaging is the prime responsibility of the physician recommending the procedure. On the other hand, the role of the medical physicist is calculating the absorbed dose of radiation in a diagnostic procedure that may potentially reach an unexpected conceptus. It is the duty of the radiologist to estimate the risk of these doses and find means to minimize the associated risk (14)*.* Few policies exist to determine the legal liability of both clinicians and radiologists in case of harmful radiation exposure, leaving medical professionals vulnerable to malpractice suits and patients liable to risks of radiation exposure (7). This was adequately stated by Berlin in 1996 that highlighted the absence of a governmental regulation or a national guideline, which obligates radiologists to investigate the pregnancy status of women in childbearing age before radiation exposure (15). Later, in 2000, the International Committee on Radiological Protection published a recommendation that encourages physicians to inform radiologists regarding the pregnancy status of referred patients and advises radiologists to adequately verify the pregnancy status of women before radiation exposure (2). 

***- Safety measures***

Different radiation procedures have different precautions, depending on the type of radiation and the body area to be imaged (7). There is a defined threshold radiation dose of 0.1Gy (10 rad), below which there is no practical risk of radiation-induced abortion or congenital malformations. The usual radiation dose, delivered during plain x-ray imaging, is usually less than 0.02 Gy (2 rad), while it rises to 0.02-0.035 Gy (2-3.5 rad) during computed tomography (CT). Based on these calculations, even repeated abdominal or pelvic CT imaging should pose no theoretical risk to the fetus (16). However, the National Council on Radiation Protection and Measurements stated a principle entitled "As low as reasonably achievable" or "ALARA" which highlighted that no radiation exposure level is entirely free of risk and that the safety of the procedure should be evaluated In terms of benefit versus risk (17). In 2006, the National Academy of Sciences issued a report which highlighted the link between low levels of radiation exposure and the risk of teratogenesis and cancer induction (18). Imaging other body areas as the chest or extremities holds lower risk to the fetus as long as the woman is positioned properly and the diagnostic procedure is medically justified (19, 20). 

To further minimize fetal exposure, the 10-day and 28-day rules were introduced. They state that radiological procedures that deliver low doses to the fetus should be restricted to the first 28 days of last menstrual cycle, while those that deliver high fetal doses (> 0.01Gy to the fetus) as pelvic computed tomography (CT) and contrast radiological procedures should be restricted to the first 10 days of the menstrual cycle. These rules apply to patients with a regular cycle length of 28 days and should be modified according to cycle length (21, 22). Despite the potential benefits of applying this rule, it creates some difficulty scheduling diagnostic tests; therefore, it is no longer applied in most radiology centers (7).

**B) What to do?**

Despite the absence of a standard regulation to guide imaging testing in these situations, several respected authorities released non-regulatory national guidelines for imaging potentially pregnant women. These recommendations are primarily based on expert committee reports or the clinical experience of respected authorities (grade D recommendation; appendix B). Further studies with stronger evidence are needed to establish a higher grade guideline for imaging potentially pregnant females. Some of the available recommendations in this regard are as follow:

***- Recommendations of American College of Radiology (ACR)***

In 2008, the ACR released a national guideline for imaging potentially pregnant women (Figure 1). However, ACR strongly recommended that each institution should develop its own policy because every individual case may require modification of the highest grade guidelines (16). AAR recommendation composes different steps:

***History taking***

Obtaining history from the patient's record or direct questioning may be feasible and reliable for the inpatients. Further assessment of the reproductive status of these females can further minimize the risk of unintended fetal exposure to radiation (16).

Defining the type of the imaging procedure is essential to determine the risk it poses to a potential conceptus. Some imaging procedures result in a low level of uterine exposure that the decision to proceed with the imaging test is not influenced by the pregnancy status. These procedures include radiographic imaging of the head, the chest (with the possible exception of the third trimester), and extremities (18, 22). Performing mammography is not contraindicated during pregnancy (23). In these cases, determining pregnancy status as a routine part of medical history is recommended through direct questioning of the patient (16).

Obtaining information from patients in the reproductive age can be performed through direct verbal questioning or written formats. The advantages of written formats include standardizing the questions and further benefits of documentation (7). Questions should extend beyond pregnancy status to assess the menstrual and reproductive history of the female because this may enhance the probability of detecting an unexpected pregnancy (16). The last menstrual period should have been completed within 4 weeks from the radiological examination because radiation exposure within this period holds no significant risk to the embryo (24).

The use of contraceptive methods should not rule out pregnancy. While contraceptive use decreases the probability of pregnancy,the efficacy of the used method is a matter of professional judgment. Therefore, if doubt exists, these guidelines should be followed (14, 16).

***Pregnancy testing***

Some radiological tests are associated with a high level of radiation exposure to the uterus as direct imaging of the pelvis, abdominal/pelvic computed tomography, hysterosalpingography, and diagnostic/ interventional pelvic angiography (25). In these cases, documenting the pregnancy status, preferably through urine pregnancy test (within 72 hours before the imaging), is strongly recommended (26, 27). 

The results of a urine pregnancy test should be interpreted carefully. A positive result may require delaying, modification or cancelling of the procedure as long as no emergency state is present. On the other hand, a negative result should not substitute verbal or written questioning of the patient about her menstrual history and possibility of pregnancy (16). In case of high-risk procedures, external monitoring of radiation dose, using monitors placed around the patient's pelvis, should be considered. Documentation of the results may be helpful in planning future imaging procedures (14).

While positive pregnancy tests are useful in directing further justification, negative pregnancy tests (performed before the period is due) should be interpreted carefully. In particular, a negative urinary pregnancy test, taken at the point of care, should be confirmed with a more sensitive laboratory test (16, 28).

***Final decision***

If pregnancy could be excluded through the earlier steps, a medically indicated radiological procedure can be performed. If pregnancy is established, the patient should be informed and the clinician should be consulted about delaying, modifying or cancelling the test upon reviewing the justification for radiological procedure and assessment of the possible risk versus the desired benefit (16).

However, if uncertainty prevails, the medical physicist should consult the radiologist and the clinician to determine the best management plan according to the protocol of the host institution. If the situation is urgent, the clinician should wave pregnancy screening and the physicist should document the emergency condition that indicated waiving the test in the patient record (14).

***- Recommendations of European community of ionizing radiation exposure***

The European Community of Medical Ionizing Radiation Protection released its guidelines for protecting women of childbearing age in 2002, with a special amendment in 2007. The main headings of this recommendation are:

- In case of a female in childbearing age, the clinician and radiologist should ask the patient directly if she is pregnant.

- If pregnancy cannot be excluded, the female should be treated as pregnant.

- If there is an emergency indication for imaging, justification of radiation exposure should be documented and optimization of exposure of the mother and her fetus should be considered (24, 29, 30).

**C) Special situations**

***- Pregnancy screening in adolescent girls***

In case of pregnancy testing in unmarried girls under the age of 18 years, whose healthcare is the responsibility of the parent or the guardian (7), the radiologist must obtain the parent/guardian's consent to assess the pregnancy status of his/her minor (31). In case of approval, the radiologist can directly ask the girl about her menstrual history or pregnancy status (32). The difficulties associated with questioning minors about their pregnancy status should be addressed by a local protocol that considers associated legal issues (14, 16).

***-Radiological testing in emergency situations***

If the medical condition is urgent, the clinician should wave the screening and the justification for this waiver should be documented (33). Radiological examination of anaesthetized patientsshould be determined by a local institutional policy where pregnancy condition is established before anesthesia (16). Other cases that may require waiving pregnancy screening may include sexually inactive females and females with infertility (14).

**D) Managing missed cases**

If a patient went through a radiological test while being pregnant, the clinician should counsel the patient regarding the event and perform a prospective risk assessment (16). As the possible effects depend on the absorbed dose and conception age, these data need to be known to offer an accurate risk assessment. Although most radiological examinations pose a small risk to the fetus, the patient should be informed in a non-alarming way about the possible risks of radiation (14). In case of exposure to a dose exceeding 0.1Gy (10 rad) in the first 15 weeks of pregnancy, counseling regarding the risks of radiation exposure during pregnancy is a must (11). Otherwise, the woman should be advised to seek standard obstetric care.

**E) Administration of contrast media**

Low molecular weight contrast media cross the human placenta and appear in the fetal circulation few minutes after maternal injection with clinical doses. Interestingly, the ACR does not recommend routine pregnancy screening before the use of iodinated contrast media, which are classified by the FDA as category B medications (34). Although no case of teratogenesis have been recorded with gadolinium based substances, the ACR recommends using gadolinium contrast media with caution at the lowest diagnostic doses, and only when critically justified (34, 35). 

## Conclusion:

Pregnancy screening before radiation exposure in reproductive age females is essential. Establishing an international regulation or standard protocols is essential to guide clinicians and radiologists through the appropriate interventions to detect pregnancy in these situations. In the light of the current evidence, urine pregnancy testing should not be performed in every female, applying for radiological examination.
